# Gross Motor Development by Age and Functional Level in Children with Cerebral Palsy from 6 Months to 17 Years—A Norwegian Population-Based Registry Study

**DOI:** 10.3390/jcm14010178

**Published:** 2024-12-31

**Authors:** Reidun Birgitta Jahnsen, Harald Weedon-Fekjar, Gerd Myklebust, Gunfrid Vinje Storvold

**Affiliations:** 1 Norwegian Quality and Surveillance Registry of Cerebral Palsy, Oslo University Hospital, 0424 Oslo, Norway; gerd.mykl@gmail.com; 2 Institute of Health and Society, University of Oslo, 0313 Oslo, Norway; 3 Oslo Center for Biostatistics and Epidemiology, Oslo University Hospital, 0424 Oslo, Norway; harald.weedon-fekjar@medisin.uio.no; 4 Regional Centre for Habilitation, Department of Mental Health, Norwegian University of Science and Technology (NTNU), 7491 Trondheim, Norway; gunfridvinje.storvold@helse-nordtrondelag.no; 5 Department of Child Habilitation, Levanger Hospital, Nord-Trøndelag Health Trust, 7601 Levanger, Norway

**Keywords:** cerebral palsy, early testing, motor development curves, reference percentiles

## Abstract

**Background:** Cerebral palsy is a complex lifespan disability caused by a lesion to the immature brain. Evaluation of interventions for children with cerebral palsy requires valid and reliable outcome measures. Motor development curves and reference percentiles for The Gross Motor Function Measure (GMFM-66) are valuable tools for following, predicting, comparing, and evaluating changes in gross motor skills. The aims of this study were to create motor development curves with reference percentiles based on Norwegian data and compare them with published counterparts for Canadian children aged 2–21 years. **Method:** Prospective population-based cohort data from the Norwegian Quality and Surveillance Registry for Cerebral Palsy (NorCP) for 1206 children with 3612 GMFM-66 tests between 0.5 and 17.3 years of age. Median development by Gross Motor Function Classification System (GMFCS) levels was estimated using a generalized additive regression model with smoothed parameters for location, scale, and shape (based on the R GAMLSS library). To adjust for repeated individual measurements, we report the median curve of 100 random samples with only one observation per observed child. **Results:** The Norwegian motor development curves for GMFCS levels I–IV increase up to 7 years of age before flattening off, while GMFCS level V curves are relatively flat. Overall, both motor development curves and GMFM-66 percentiles are very similar to Canadian counterparts. **Conclusions:** The existing Canadian reference curves are valid also for Norway, working well for both clinical and research applications. However, Norwegian percentiles can be used from an earlier age.

## 1. Introduction

Cerebral palsy (CP) is a lifelong motor disorder caused by an early-onset non-progressive brain lesion. Symptoms and severity vary and may cause changing activity limitations and participation restrictions throughout the lifespan, also due to associated impairments, such as cognitive, sensory, communicative, and behavioral challenges [1]. However, activity limitations in gross motor function are a core symptom in CP [2], which may interfere with the children’s possibilities to explore and interact with their environment [3,4]. Therefore, valid and reliable tools to monitor, predict, compare, and evaluate change in gross motor function in children with CP are needed. The motor development curves [2,5] and reference percentiles for the Gross Motor Function Measure (GMFM-66) [6] have shown to be valuable tools in that respect [6,7]. However, cross-national validation of the Canadian curves is not available in Norway.

The motor development curves [2] describe the relationship between age and gross motor function based on GMFM-66 scores and show the estimated average pattern of gross motor development, including both the rate of development and the limit of ability for children 2–21 years old for each of the Gross Motor Function Classification System (GMFCS) levels [2,6]. GMFM-66 scores typically peak at about 7–8 years of age before declining for GMFCS levels III–V [6].

Motor development curves have also been established in The Netherlands [8], Australia [9], and Sweden [10], with only slight deviations from the original curves presented by Rosenbaum et al. (2002) [2]. No evidence supporting a decline in gross motor function in any of the five GMFCS levels was found. However, the newly published study of motor development curves in a low-to-middle-income area of India, Brien et al. found both a slower rate and lower limit than in the aforementioned high-income countries [11].

The motor development curves made it possible to evaluate children’s gross motor function relative to the average for their age and GMFCS level. With the introduction of the reference percentiles for the GMFM-66 [6], one could monitor the gross motor development within GMFCS levels. The reference percentiles for the GMFM-66 can be used clinically as a supplement to GMFM-66 total scores. The GMFM-66 total scores measure the children’s motor capacity and can tell if the child has learned new gross motor skills. The reference percentiles, on the other hand, can tell if the change is larger or smaller than expected for children at the same GMFCS level and age.

The reference percentiles are also a very useful outcome measure in intervention research studies [4,12]. Furthermore, our research group used the reference percentiles in cohort studies investigating factors associated with enhanced gross motor progress [13]. In 2019, reference percentiles for the GMFM-66 were created in Germany [14]; however, not within GMFCS levels. In contrast to the reference percentiles by Hanna et al., [6]. Duran et al. found only a very small decline in the higher GMFCS levels and ages [14].

By using longitudinal long follow-up data for children with CP aged 6 months to 17 years from the Norwegian Quality and Surveillance Registry (NorCP) [15], the aims of this study were to create:

1. Motor development curves and compare them with published curves by Rosenbaum et al. (2002) [2].
2. Reference percentiles for the GMFM-66 and compare them with the Canadian curves reported by Hanna et al. (2008) [6].

## 2. Materials and Methods

### 2.1. Design

The study was a prospective population-based cohort design with longitudinal data from NorCP, based on a large number of GMFCS and GMFM-66 measurements.

### 2.2. Population

All children with CP registered in the NorCP who have been tested with GMFM-66 are included in the study. The registry was established in 2006, including children with CP born from 2002 onward, currently covering 96% of the population with CP in Norway [15].

### 2.3. Outcome Measures

The Gross Motor Function Classification System Expanded and Revised (GMFCS E&R) [16] classifies gross motor function in children and adolescents with CP on a five-level ordinal scale. Children at level I have minimal limitations in gross motor function and walk without restrictions at 6 years of age, while children at level V are transported in a wheelchair and have severe limitations in head and trunk control and self-mobility [5,16,17]. The GMFCS levels appear to be relatively stable over time and after interventions and can predict gross motor function up to adult age in persons with CP [2,8,18,19,20]. Change associated with different types of interventions will, in general, occur within the GMFCS level the child was assigned to rather than through a change in level [16]. GMFCS has proven valid and reliable for children and adults with CP in different cultures [5,16,19,21]. In the current study, as in NorCP, the Norwegian translation of GMFCS E&R were used [22,23].

The Gross Motor Function Measure 66 (GMFM-66) is a standardized observation instrument designed to measure gross motor function in children with CP in a specific test situation without the use of any mobility devices or orthoses. The test is shown to be valid for evaluating changes in gross motor function over time and after interventions [7]. GMFM-66 is developed through Rasch analysis of GMFM-88 [24] and has an interval-level scale ranging from 0 (lowest motor capacity) to 100 (highest motor capacity) [24]. GMFM-66 raw scores are converted to total scores using the Gross Motor Ability Estimator 2 (GMAE-2).

### 2.4. Procedure

The GMFCS E&R classification and GMFM-66 tests were performed by physiotherapists at 21 habilitation centers, all familiar with assessing gross motor function in children with CP. The introduction of GMFM-66 in NorCP was organized by face-to-face interactive workshops at all habilitation centers nationwide, and courses have been given regularly since the start in 2006. The current study used the Norwegian translation of GMFM-66 score sheets [25]. In the NorCP protocol, a yearly test with GMFM-66 is recommended; however, the follow-up assessments were performed at different intervals, and thus the number of assessments for each child varied. GMFM-66 total scores were obtained using GMAE-2.

### 2.5. Statistical Analyses

Median development by GMFCS levels was estimated using a generalized additive regression Model with smoothed parameters for Location, Scale and Shape (GAMLSS) [26]. Estimation was performed using the R GAMLSS [27,28] library’s function LMS (https://cran.r-project.org/web/packages/gamlss/, accessed on 1 February 2022) with default parameters. This implies normal distributed variation scaled by smoothed parameters for location (median), scale (variation), and shape (skewness expressed as a Box–Cox power). Smoothing was performed using cubic splines, with the degree of smoothing decided by stepwise forward and backward selection using a generalized Akaike Information Criterion (GAIC) equalling 2. To adjust for repeated individual measurements, we report the median curve of 100 random samples with only one observation per child.

In the Supplementary File S1, “Modelfit-DevCP.pdf”, a Q–Q plot of model residuals (using all data), plus plots of residuals by age with a smoothed line for the mean (using Friedman’s Super Smoother, StataCorp, Boston, USA) are shown. The residuals are normally distributed with only minor departures around the edges of the observed age range. The variation that varies by age is taken into account by the “gamlss” modelling.

When comparing reference curves across countries, we evaluated the uncertainty by 1000 bootstrap calculations. For each bootstrap sample, we calculated reference curves based on eight random samples for each country with only one observation per child and used the mean difference of all pairs across the two countries. All analyses and plots were performed using the R statistical software package 4.4.0 and GMLSS version 4.4-22.

### 2.6. Ethics

The United Nations Convention on the Rights of Persons with Disabilities (CRPD) [29] emphasizes the right to appropriate and accessible healthcare for people with disabilities. However, even in high-income countries, many individuals with disabilities experience the healthcare system as fragmented, uncoordinated, short-sighted, reactive, and non-accessible [29]. The aim of NorCP, which is both a national medical quality registry and a surveillance program, is to contribute towards preventing secondary complications in individuals with CP by a standardized and systematic follow-up at predictable intervals and thus enhance equal health services in the whole country and reduce unwanted variation in services. The Regional Ethical Committee for research in medicine and health science (REC) concluded that the project fell outside the scope of the Health Research Act, cf. Section 2, and could therefore be carried out without approval by REC (REC south-east 2013/1527).

## 3. Results

This study included 1206 children and teenagers with 3612 GMFM-66 tests. The mean age is 5 years and 8 months, and 482 (40%) are girls. Their age at the time of the tests ranged from 6 months to 17 years and 4 months. The number of tests ranged from one to 14 per child, with a mean of 3 tests per child (Table 1). The distribution of CP subtypes showed that 537 (45%) children had spastic unilateral CP, 516 (43%) had spastic bilateral CP, 95 (8%) had dyskinetic CP, 40 (3%) had ataxic CP, and 18 (1%) had unspecified CP. In all, 631 children (52%) were classified at GMFCS level I, 198 (16%) at level II, 108 (9%) at level III, 128 (11%) at level IV, and 141 (12%) at level V (Table 1).

**Figure 1:**
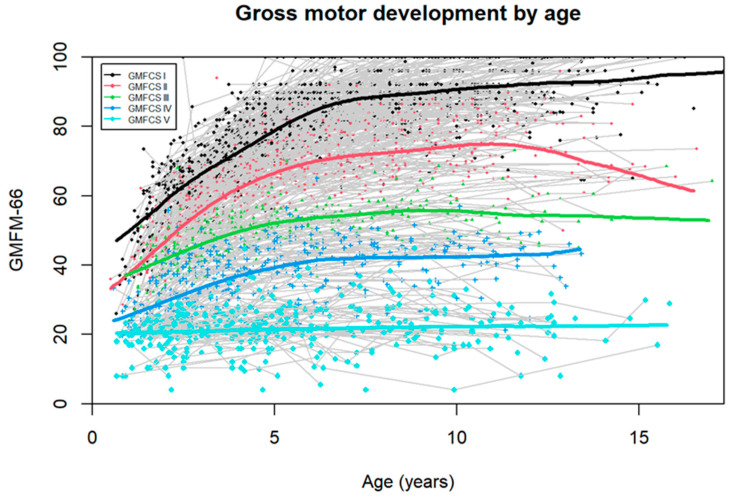
Estimated Norwegian motor development curves based on observed GMFM-66 by GMFCS level, given as median values from smoothed GAMLSS regression.

The estimated Norwegian motor development curves increase until around 7 years of age before flattening off, except for GMFCS V, which shows no change by age (Figure 1).

**Figure 2:**
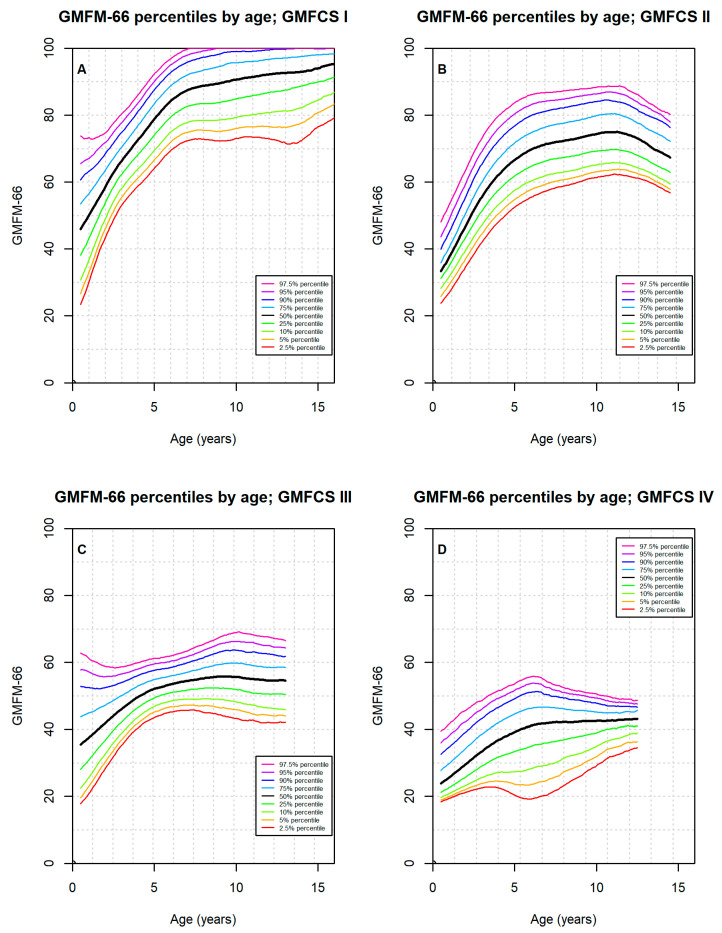

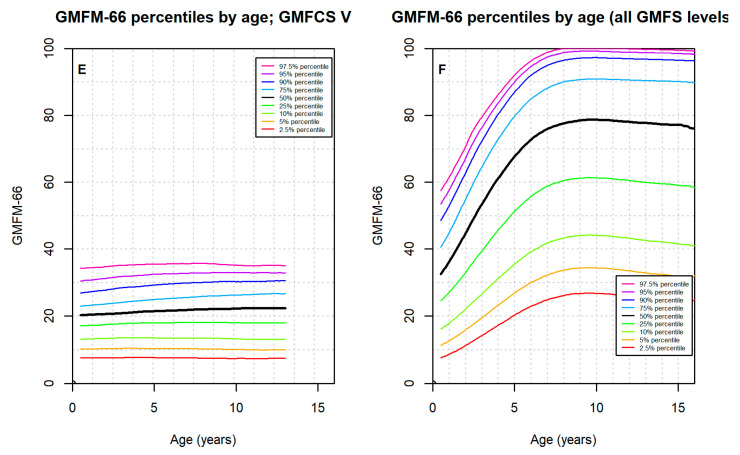
Estimated GMFM-66 percentiles by GMFCS levels in 1206 Norwegian children.

The Norwegian reference percentiles for each GMFCS level and for all GMFCS levels together by age are presented in Figure 2. There is a considerable variation in each GMFCS level, which must be taken into account in the interpretation of clinical results.

**Figure 3:**
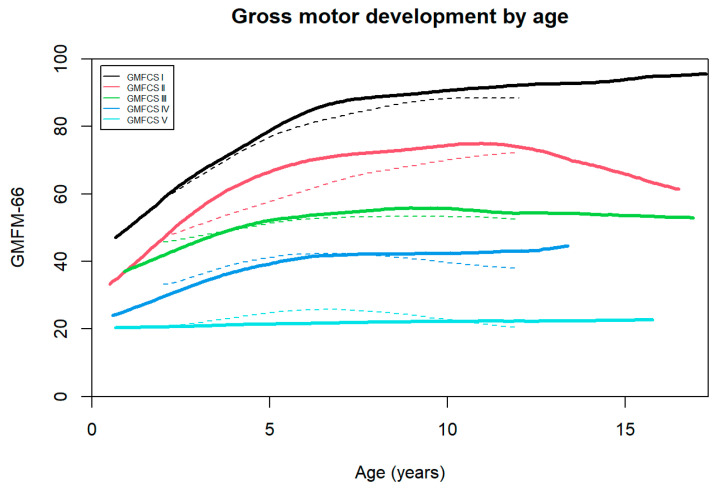
Estimated Norwegian median GMFM-66 by age by GMFCS levels (solid lines) compared to Canadian reference data (dashed lines).

The Norwegian developmental curves are highly similar to the Canadian motor development curves by Rosenbaum et al. (2002) [2], as all the Canadian curves fall within the 95% confidence intervals (CI) of the Norwegian curves (Figure 3).

**Figure 4:**
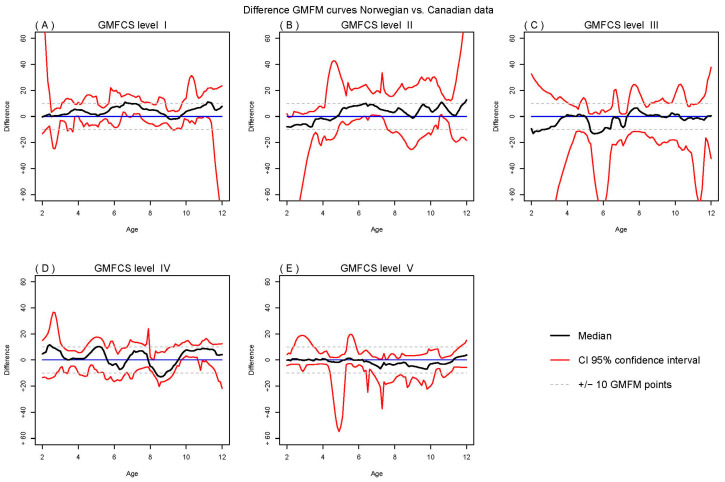
Mean difference (black line) between the Norwegian reference percentile curves and the earlier published Canadian reference curves with 95% confidence interval limits (red lines).

The 95% confidence intervals of the Norwegian (mean) reference curves cover the Canadian reference curves almost entirely (Figure 4), indicating that all the difference might be random. We could not take into account the uncertainty of the Canadian data due to lack of data access.

## 4. Discussion

### 4.1. Summary of Findings

In this large population-based cohort study based on longitudinal data from NorCP, we created motor development curves describing the estimated average pattern of gross motor development for children aged 6 months to 17 years and 3 months for each of the GMFCS levels. The Norwegian curves are almost identical to the Canadian development curves presented by Rosenbaum et al. (2002) [2].

In addition, reference percentiles for the GMFM-66 for the same population were created and compared with the reference percentiles presented by Hanna et al. (2008) [6]. With the exception of GMFCS level III, which started somewhat lower but reached the same levels as the Canadian percentiles, we found that the Norwegian and Canadian reference percentiles were largely similar. We did not find the tendency for decline in percentiles at GMFCS level IV and V as seen in the Canadian curves, but rather a tendency for decline at level II. This decline was, however, not statistically significant, evaluated by comparing the estimated median at 10 and 12 years of age using all combinations across 100 bootstrap replications.

### 4.2. Are the Motor Development Curves Published by Rosenbaum et al. (2002) [2] Valid for Children and Youth with CP in Norway?

As expected, our findings confirm the presence of five distinct gross motor trajectories according to GMFCS levels, consistent with prior research conducted in Canada [2,19], The Netherlands [8], Australia [9], and Sweden [10]. The statistical approach employed in the Canadian, Dutch, and Swedish studies was the same, enabling a straightforward comparison between them. Smits et al. (2013) [8] also argue for great similarities between the Canadian and Dutch curves. However, the newly published study from India, which is a low-to-middle-income country, presents curves with a slower rate and lower limit of motor development [11]. This may indicate the relationship between socio-economic status and health and development, and thus document the importance of validating prognostic classification systems in different contexts [11].

Visual inspection of Figure 3 reveals significant resemblances between the Norwegian and Canadian curves [2], with minor deviations observed primarily within GMFCS levels II. Leveraging advanced and refined statistical methodologies in our study complicates the direct comparison of our curves with the Canadian curves. Nevertheless, the fact that the Canadian curves fall within the 95% CI band of our curves demonstrates an impressive overlap. Consequently, we contend that the applicability of the Canadian curves extends to the population of children in Norway.

Plotting GMFM-66 by GMFCS level follows the common clinical practice as seen in earlier publications and clinical GMFM software(GMAE-2) [7]. Unlike Duran et al. (2019) [14], we think that it is clinically relevant to compare a child with children at the same age and GMFCS level. GMFCS levels are shown to be predominantly stable, and improvements after interventions are most likely to occur within the assigned GMFCS level. Intervention studies have used the Canadian percentiles, and to facilitate sharing and comparing of research results, we decided to create reference percentiles for each GMFCS level as Hanna et al. did in 2008 [6] (Figure 2A–E). However, we have also provided reference percentiles for all children with CP (Figure 2F), and visual inspection shows large similarity with the study by Duran et al. (2019) [14]. Recently, a new study by Sanderlin et al. (2024) [26] showed that the informative value of one GMFM66 percentile [14] versus five GMFM66 percentiles [6] was compared with regard to the assessment of changes in GMFM66 (i.e., longitudinal evaluation) and found to be comparable.

### 4.3. Are the Reference Percentiles Published by Hanna et al. (2008) [6] Valid for Children and Youth with CP in Norway?

To our knowledge, this study is the first cross-national validation of the reference percentiles within GMFCS levels created in Canada [6]. The striking congruence observed between the motor developmental curves of Norway and Canada suggests a shared course of gross motor development among children with CP across both nations. Consequently, an analogous resemblance is anticipated in the reference percentiles for these two populations, as was indeed seen in our findings. The differences might actually only be random noise due to the limited number of children under study. However, the relative magnitude of these differences is modest, and we argue that favoring the Norwegian curves over their Canadian counterparts would lack clear justification. To facilitate international research comparability, we advocate for using the Canadian reference percentiles within the Norwegian context.

### 4.4. How Can the Reference Percentiles Be Useful in Clinical Practice and Research?

Hanna et al. (2008) [6] provide excellent examples of how to use GMFM-66 total scores and reference percentiles in combination. If a child has learned new gross motor skills since the previous measurement (an increase in GMFM-66 total scores), reference percentiles can tell whether that particular gain is more or less than expected for a child at that GMFCS level and age. For example, a change of 2 in GMFM-66 total scores over a 6-month period can be more than expected for 7-year-old children in GMFCS levels III, IV, and V, as much as expected for a 3-year-old child in GMFCS level II, and less than expected for a 3-year-old child in GMFCS level I.

Reference percentiles are also important in research, as a statistically significant increase in GMFM-66 total scores following an intervention can be larger than expected (an upward shift in percentiles), suggesting an effective intervention. However, sometimes an increase in GMFM-66 total scores can be as expected (same percentile) and suggest that the intervention’s effectiveness parallels standard care, or the change in GMFM-66 score can even be less than expected (a downward shift in percentile), which might signal that the effects of the intervention are inferior to standard care. Moreover, as a slight drop in GMFM-66 scores is expected for some older children [6], an intervention resulting in unchanged GMFM-66 scores could in fact demonstrate effectiveness, manifested as a percentile increase.

As the present study shows that the reference percentiles presented by Hanna et al. (2008) [6] are also valid for Norwegian children, we can continue to use them both in clinical practice and in research. A study by Elvrum et al. (2024) confirms that the Norwegian percentiles can be used from an earlier age than the Canadian ones, which start at 2 years of age [30].

### 4.5. Limiatations

The age range discrepancy should not be a problem, as we only compare the curves in the aged range with overlapping data. Theoretically, a steep change just after our observation period could impact the smoothed lines, but this is highly unlikely, and we find no such change in the reported Canadian comparison data. Even if the present study includes a large number of children, the results would have been strengthened with more children in the older age groups and in the higher GMFCS levels. Our suggestion for further research would be an international collaboration between all the mentioned countries. Since our results are so similar, the results would be even more valid and reliable if we merged all the data and conducted an international study with development curves and reference percentiles, both for each GMFCS level and for all the children together.

## 5. Conclusions

The current study demonstrates an impressive overlap between the Norwegian and the Canadian motor development curves. Consequently, we contend that the applicability of the Canadian curves extends to the population of children in Norway. Our results also indicate that the Norwegian reference percentiles are sufficiently similar to the Canadian percentiles that the Canadian curves can be used in clinical work and in research. However, for children below two years of age, the Norwegian would be more appropriate.

## Figures and Tables

**Table 1:** Study population characteristics.

GMFCS Level	N	Girls (%)	Age Range (Years)	GMFM-66 Scores	Mean Observations per Individual
I	631	41	0.7 to 17.3	22 to 100	2.8
II	198	41	0.5 to 16.6	23 to 96	3.4
III	108	39	0.9 to 17	22 to 73	3.1
IV	128	38	0.6 to 13.4	4 to 65	3.1
V	141	39	0.7 to 15.8	4 to 45	2.9
All	1206	40	0.5 to 17.3	4 to 100	3.0

## Data Availability

Data from this study can be made available upon request to the corresponding author.

## Supplementary Materials

The following supporting information can be downloaded at: https://www.mdpi.com/article/10.3390/jcm14010178/s1, Figure S1: Modelfit-DevCP.pdf.
