# Supplementary material for: Gross Motor Development by Age and Functional Level in Children with Cerebral Palsy from 6 Months to 17 Years—A Norwegian Population-Based Registry Study
Source: J Clin Med. 2024 Dec 31;14(1):178. doi: 10.3390/jcm14010178 (PMC11721768; doi:10.3390/jcm14010178)
Supplement: Supplementary file 1 [file jcm-14-00178-s001.zip › jcm-3337892-supplementary.pdf]

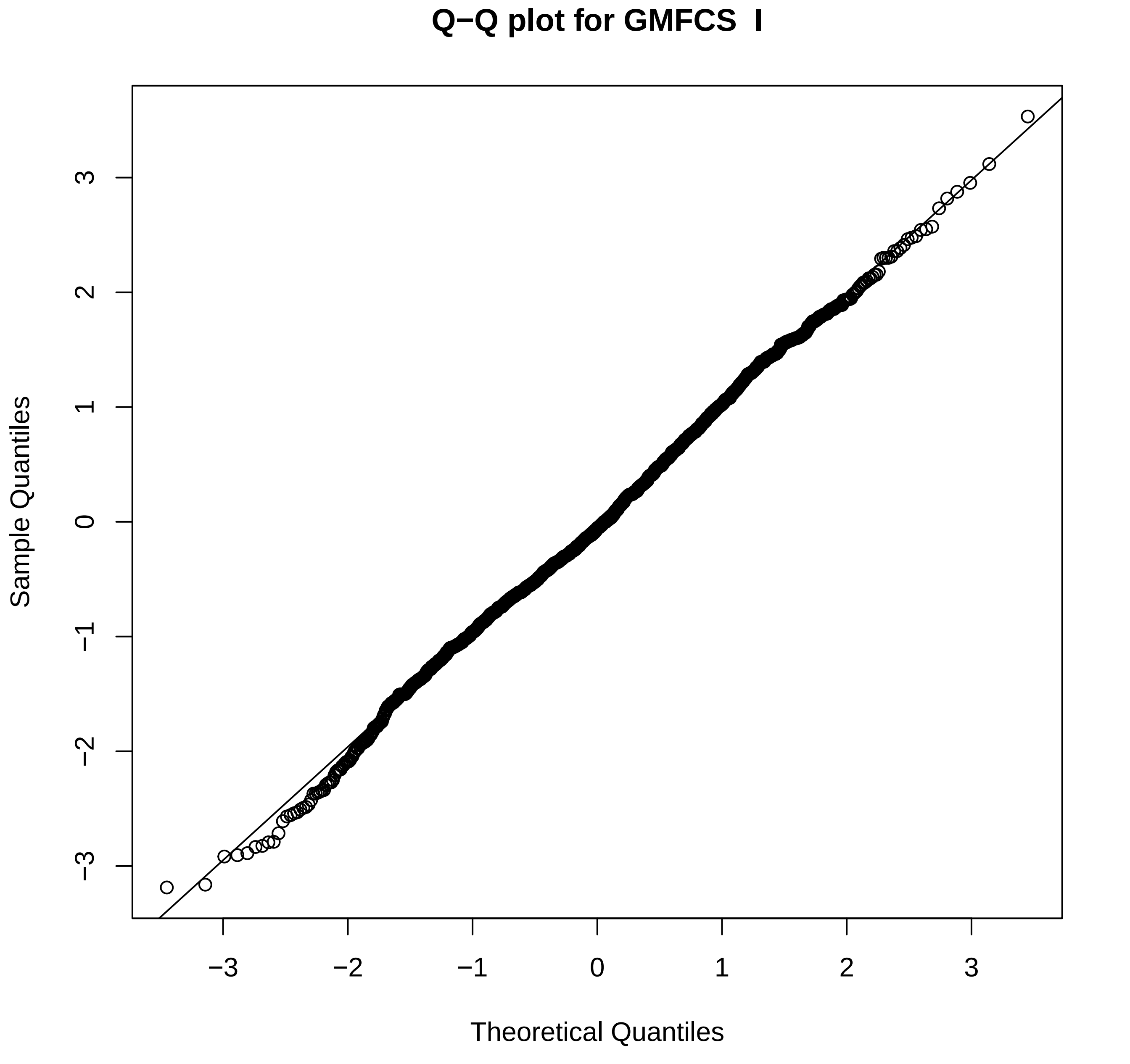

Q−Q plot for GMFCS I
Sample Quantiles
Theoretical Quantiles
−3
−2
−1
0
1
2
3

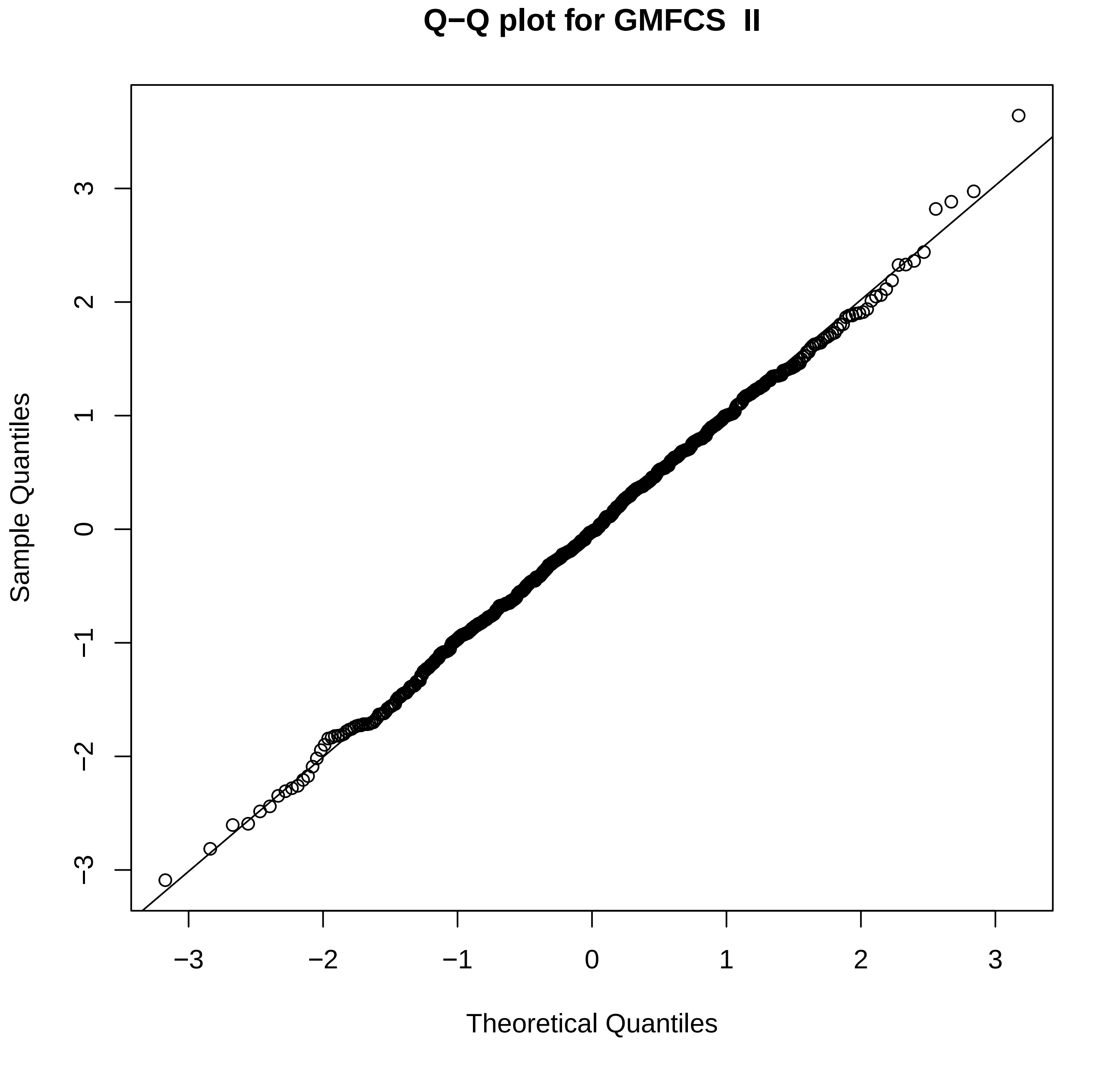
Q−Q plot for GMFCS II
Sample Quantiles
Theoretical Quantiles
−3
−2
−1
0
1
2
3

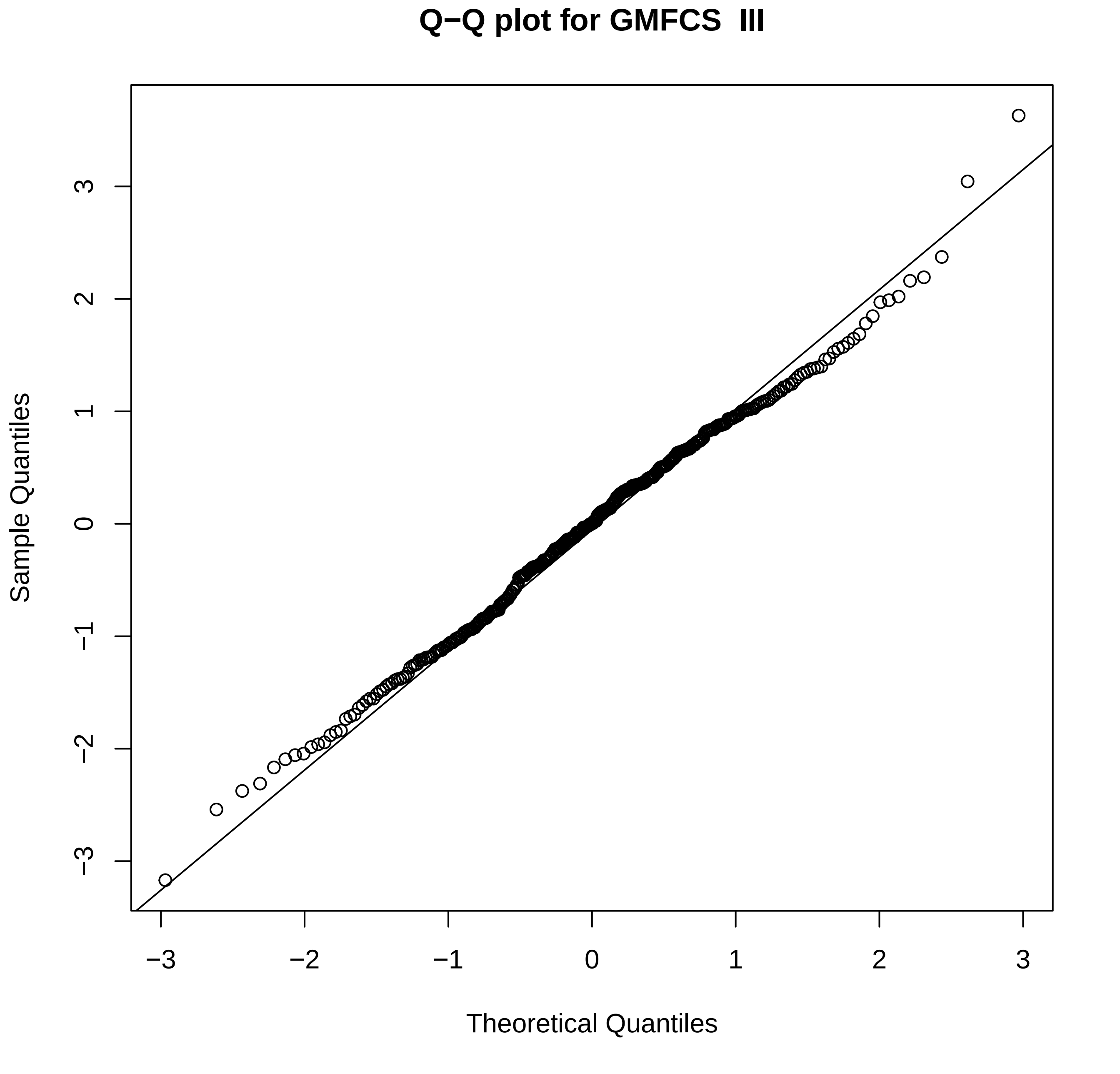
Q−Q plot for GMFCS III
Sample Quantiles
Theoretical Quantiles
−3
−2
−1
0
1
2
3

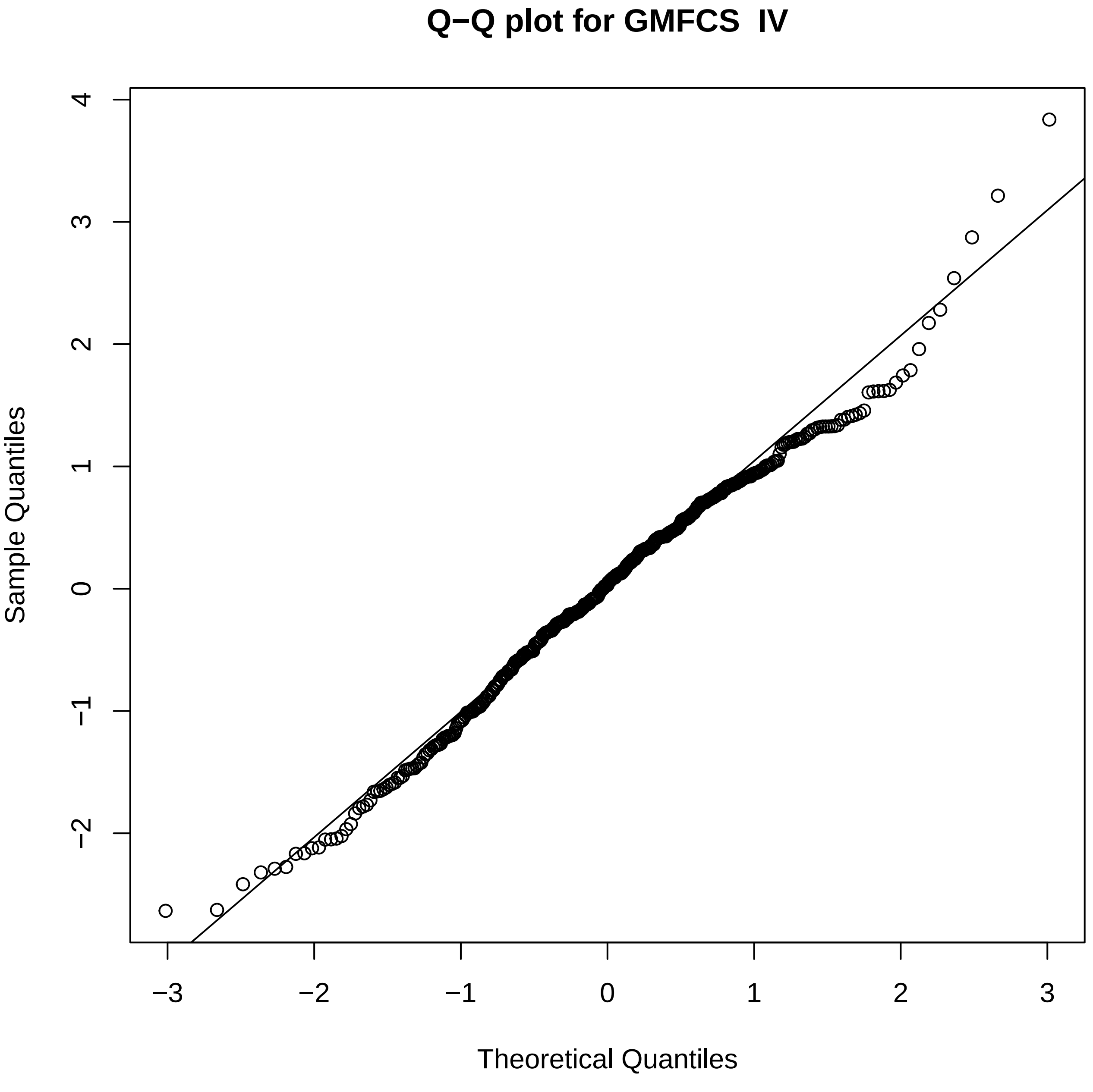
Q−Q plot for GMFCS IV
Sample Quantiles
Theoretical Quantiles
−3
−2
−1
0
1
2
3
4

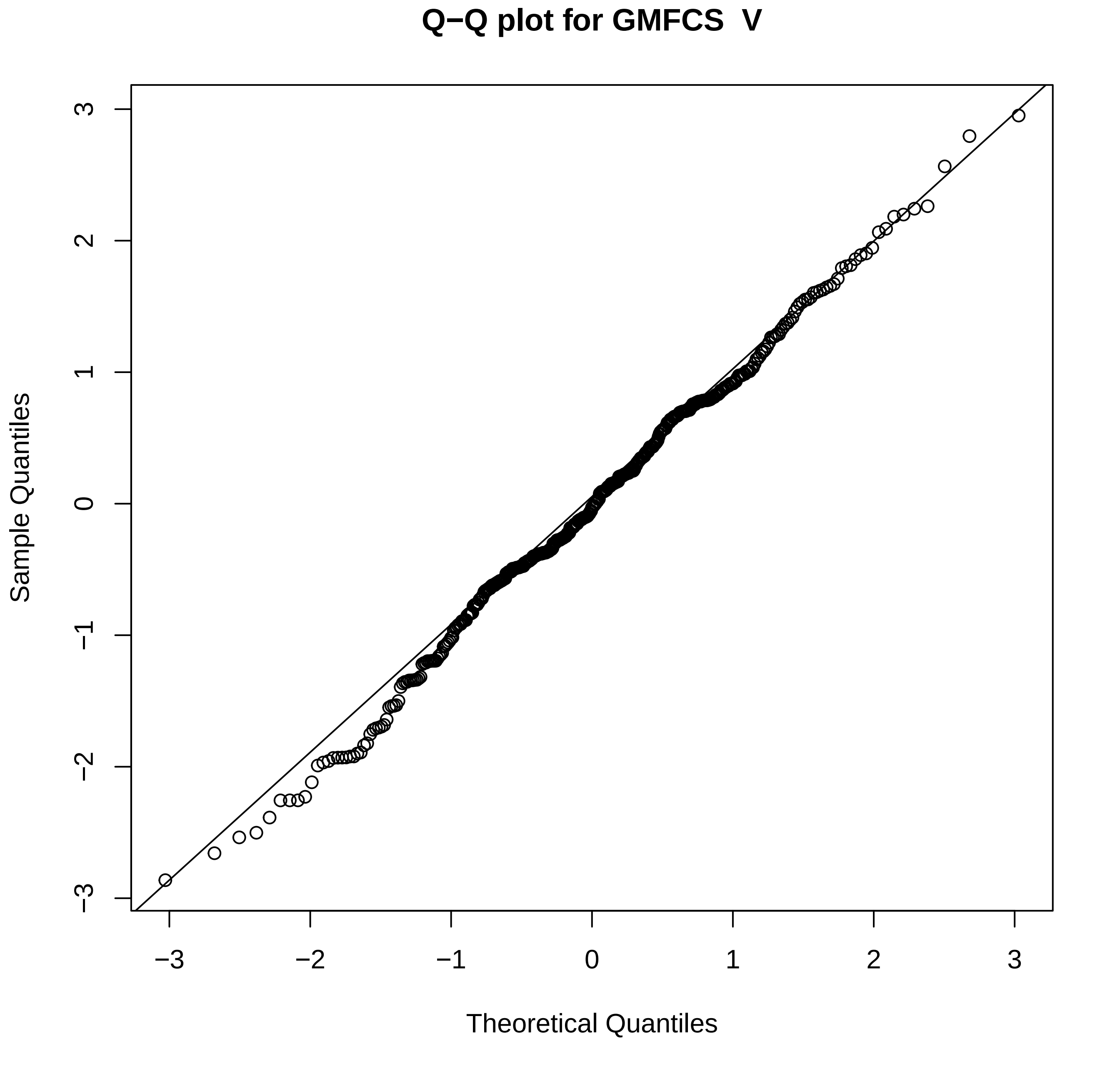

Q−Q plot for GMFCS V
Sample Quantiles
Theoretical Quantiles
−3
−2
−1
0
1
2
3

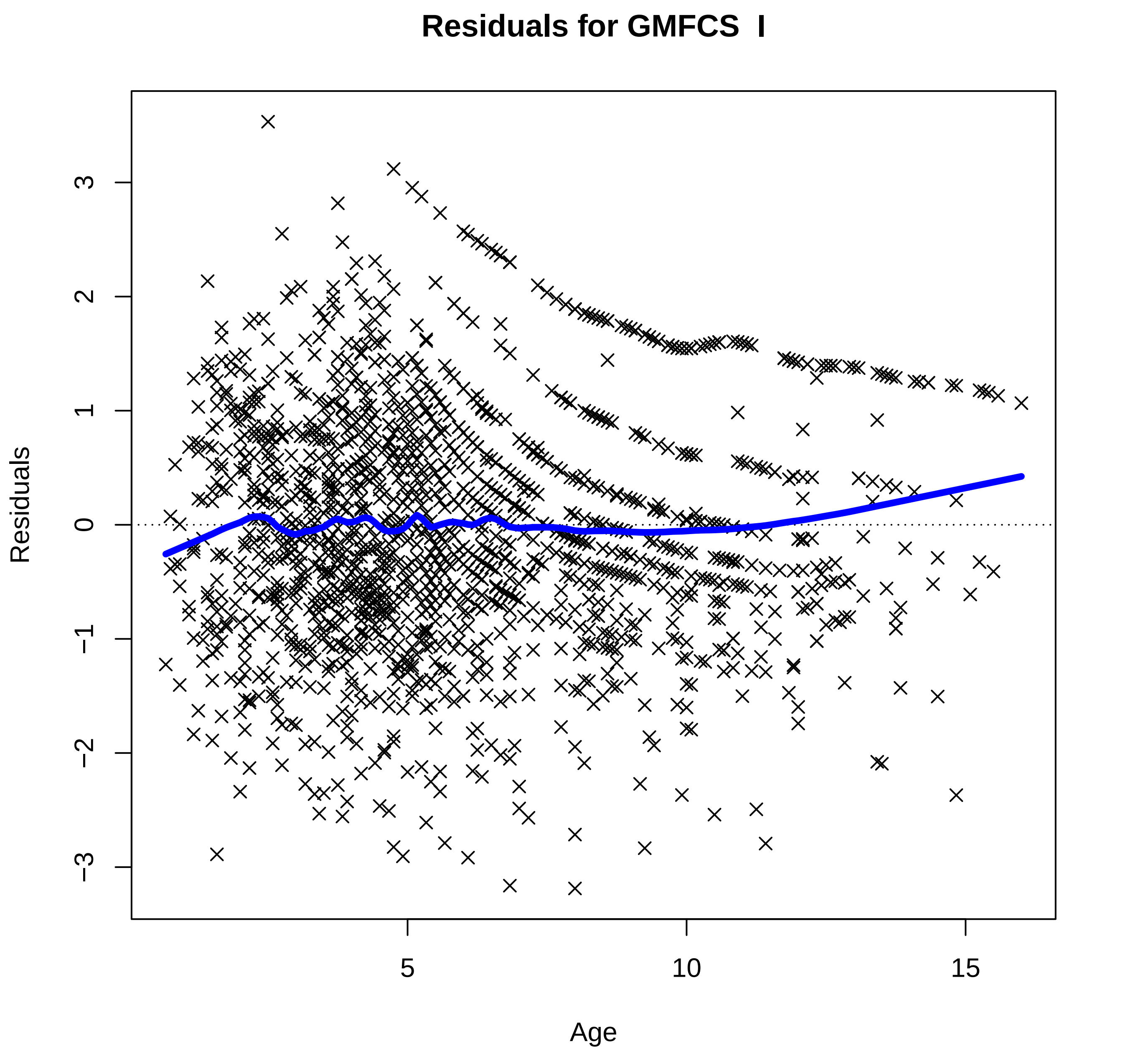
Residuals for GMFCS I
Residuals
Age
3
2
1
0
−1
−2
−3
5
10
15

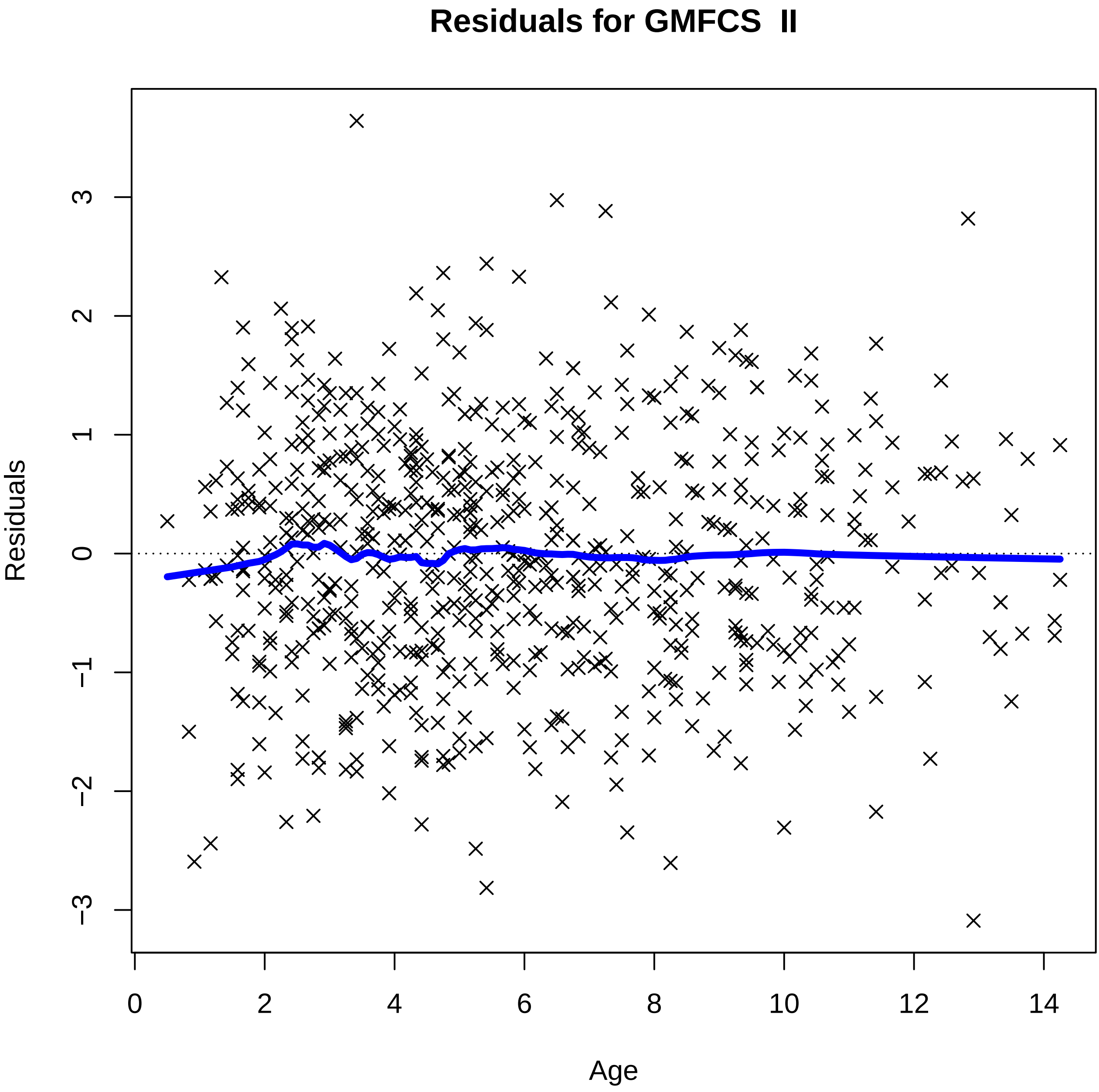
Residuals for GMFCS II
Residuals
Age
3
2
1
0
−1
−2
−3
0
2
4
6
8
10
12
14

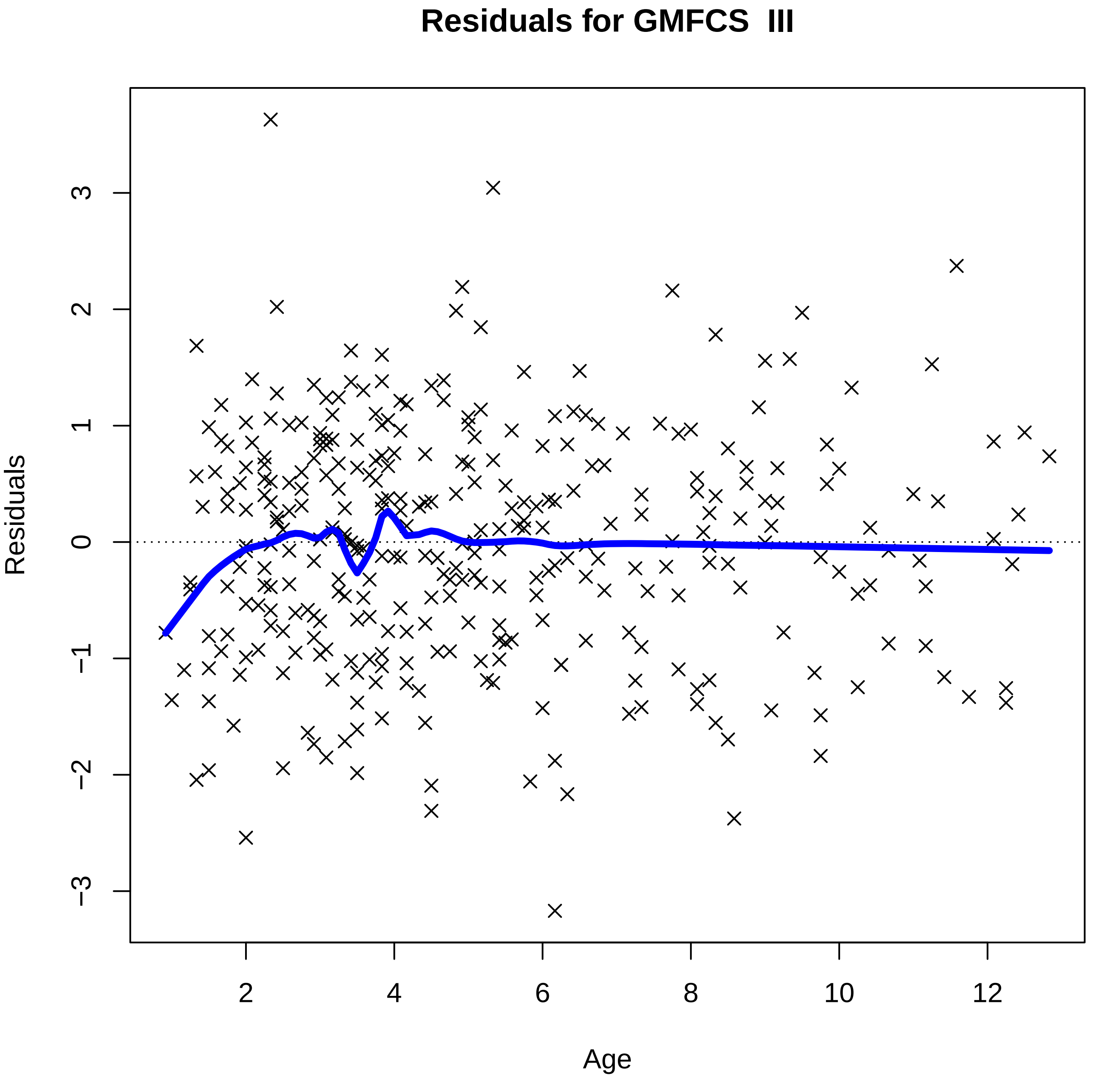

Residuals for GMFCS III
Residuals
Age
3
2
1
0
−1
−2
−3
2
4
6
8
10
12

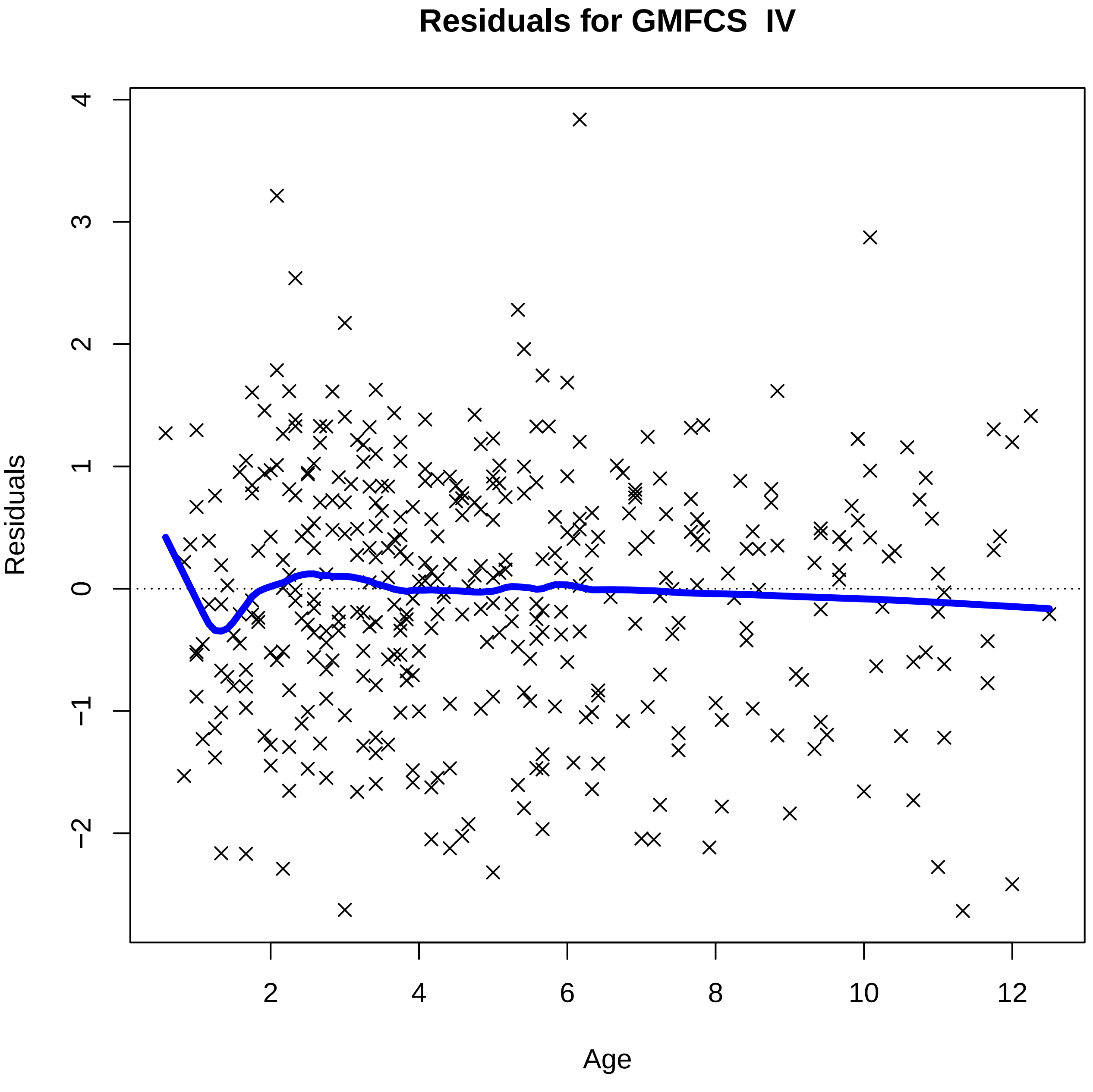

Residuals for GMFCS IV
Residuals
Age
4
3
2
1
0
−1
−2
2
4
6
8
10
12

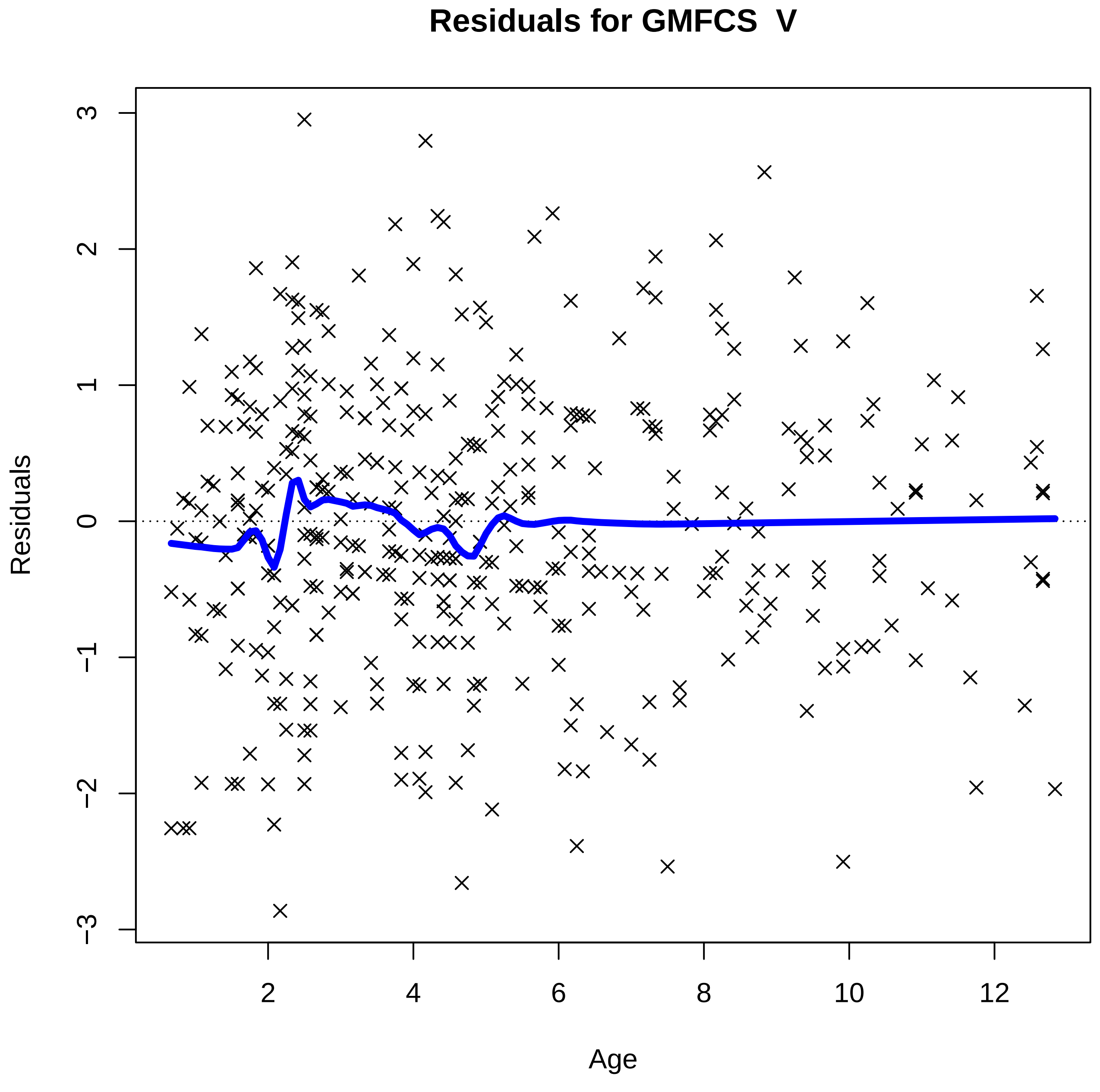
Residuals for GMFCS V
Residuals
Age
3
2
1
0
−1
−2
−3
2
4
6
8
10
12
